# Comprehensive analysis for diagnosis of preoperative non-invasive follicular thyroid neoplasm with papillary-like nuclear features

**DOI:** 10.1371/journal.pone.0218046

**Published:** 2019-07-05

**Authors:** Hye Seung Lee, Jae-Wook Lee, Ji Hyun Park, Wan-Seop Kim, Hye Seung Han, Seung Eun Lee

**Affiliations:** 1 Department of Pathology, Konkuk University Medical Center, Konkuk University School of Medicine, Seoul, Republic of Korea; 2 Department of Radiology, Konkuk University Medical Center, Konkuk University School of Medicine, Seoul, Republic of Korea; 3 Department of Hemato-Oncology, Konkuk University Medical Center, Konkuk University School of Medicine, Seoul, Republic of Korea; Universidade do Porto Faculdade de Medicina, PORTUGAL

## Abstract

**Objective:**

The current paradigm in the treatment of patients with non-invasive follicular thyroid neoplasm with papillary-like nuclear features (NIFTP) is a diagnostic lobectomy rather than complete thyroidectomy and postoperative radioiodine treatment. Consequently, preoperative diagnosis of NIFTP is considered to be important.

**Methods:**

We performed the comprehensive analysis for diagnosis of preoperative 20 NIFTPs in comparison with 41 invasive encapsulated follicular papillary thyroid carcinomas (I-EFVPTCs) using the Korean Thyroid Imaging Reporting and Data System (K-TIRADS), Bethesda System for Reporting Thyroid Cytopathology (TBSRTC), and molecular analysis for *BRAF* and *RAS* mutations.

**Results:**

K-TIRADS 3 was identified as the most common sonographic diagnosis in both NIFTP and I-EFVPTC. Unlike I-EFVPTC, K-TIRADS 5 was not identified in NIFTP. AUS/FLUS was the most common cytopathological diagnosis and none of the cases were classified as malignant category in both groups, although the difference in distribution was not significant between the groups. *BRAF* mutation was not found in NIFTP but was present in 9.8% of cases in I-EFVPTC. The frequency of *RAS* mutation in I-EFVPTCs was twice as high as that of NIFTP. Wild-type *BRAF* and *RAS* in NIFTP was significantly higher than I-EFVPTC.

**Conclusion:**

The existence of overlapping features between the groups was evident, hence conclusive distinction between radiology, cytology and molecular analysis could not be achieved. Apparently, the diagnosis of NIFTP based on comprehensive analysis was not confirmable but could perceive or at least favor the diagnosis of NIFTP.

## Introduction

Follicular variant of papillary thyroid carcinoma (FVPTC) is the second most common variant of papillary thyroid carcinoma (PTC) and its incidence has recently increased due to enhancement in recognition [1–3]. FVPTC is a heterogeneous disease composed of two distinct biological groups: “encapsulated/well-demarcated tumor (EFVPTC)” and “infiltrative FVPTC (IFVPTC)”. EFVPTCs have a clear demarcation with or without fibrous capsule, while IFVPTCs have an infiltrative border with tumor follicles invading between the surrounding benign follicles. The two groups have clinically and biologically distinct characteristics [4]. While IFVPTCs have significant metastatic potential, EFVPTCs lack metastatic potential or recurrence risk if angioinvasion or capsular invasion is absent. EFVPTC is also sub-classified into noninvasive and invasive EFVPTC subgroups (I-EFVPTC) based on capsular or vascular invasion [5]. Recently, Nikiforov et al. [6] reported that noninvasive EFVPTC should be termed as non-invasive follicular thyroid neoplasm with papillary-like nuclear features (NIFTP) due to very low risk of adverse outcome. Therefore, patients with NIFTP should receive lobectomy only and no postoperative radioiodine treatment. Consequently, preoperative diagnosis of NIFTP is considered to be important.

Ultrasonography (US)-guided fine-needle aspiration (FNA) biopsy is a standard diagnostic tool for preoperative evaluation of thyroid nodules. However, preoperative US features of FVPTC are challenging due to relatively benign appearance [7–9]. The Korean Society of Thyroid Radiology recently proposed a new clinically feasible risk stratification system for thyroid nodules, the Korean Thyroid Imaging Reporting and Data System (K-TIRADS) [10], based on the US patterns composed of the solidity, echogenicity, and suspicious US features based on the analysis of 2,000 nodules including 454 nodules that were pathologically proven as malignant [10]. More recently, Hahn et al. [11] demonstrated that the malignancy risk predicted by K-TIRADS was significantly higher in the non-NIFTP than in the NIFTP subgroup.

According to the The Bethesda System for Reporting Thyroid Cytopathology (TBSRTC), a concept of “indeterminate nodules” was introduced, which is including “Atypia of Undetermined Significance/ Follicular Lesion of Undetermined Significance (AUS/FLUS)”, “Follicular Neoplasm/Suspicious For a Follicular Neoplasm (FN/SFN)”, and “Suspicious for Malignancy (SM)” category [12,13]. FNA biopsy revealed that indeterminate nodules comprise about 15–25% of thyroid nodules [14]. Cytologically indeterminate thyroid nodules remain a challenge for physicians during the management of patients due to the uncertain malignancy risk [15]. Unfortunately, the majority of the histologically confirmed NIFTP cases were diagnosed as cytologically indeterminate TBSRTC category [16,17]. Molecular analysis for cytologically indeterminate thyroid nodules has contributed to an improvement in the diagnostic accuracy [18]. Genetic alterations that include *BRAF*, *RAS*, and *RET/PTC* in PTC can be preoperatively detected in FNA specimens from thyroid nodules. TCGA described that the majority of classic PTCs clustered with *BRAF* V600E-mutated tumors, whereas, *RAS* and *PAX8/PPARγ* alterations were identified in follicular-patterned thyroid tumors, including follicular adenoma, follicular carcinoma, and FVPTC [19].

Several studies have attempted to discriminate between NIFTP, I-EFVPTC, and IFVPTC by sonographic, cytopathologic, and molecular analysis [5,11,20–22]. However, few types of research have been integrated into three analyses to distinguish between NIFTP and I-EFVPTC subgroups. In the present study, we performed the comprehensive analysis for diagnosis of preoperative NIFTP in comparison with I-EFVPTC using the K-TIRADS classification, TBSRTC, and molecular analysis.

## Materials and methods

### Patient selection

The archival data were fully anonymized before the beginning of the research and were waived the requirement for informed consent by the Institutional Review Board (IRB) of Konkuk University Medical Center (KUMC), Seoul, Korea (KUH1210060). First, we identified patients who underwent thyroidectomy for thyroid nodules and were diagnosed with a follicular variant of papillary thyroid carcinoma (FVPTC) between January 2015 and October 2018 at KUMC (n = 205). The preoperative FNA specimens with postoperatively confirmed pathological diagnosis obtained from the matched patients were evaluated. Of 205 samples, 139 (67.8%) patients were EFVPTCs and 66 (32.2%) were IFVPTCs. Of 139 EFVPTCs, the samples with concurrent other variants of PTCs (n = 71) were excluded and the details are as follows; 44 with classic type, 14 with IFVPTC, 8 with classic type and IFVPTC, 2 with tall cell variant, 1 with classic type and tall-cell variant, 1 with IFVPTC and solid / trabecular variant, and 1 with classic type, IFVPTC, and tall cell variant. Additionally, 7 samples were reclassified into classic type due to true papillae also excluded after reviewing the slides. A total of 61 patients with EFVPTC were finally enrolled in this study. Electronic medical records (EMR) including clinical information, results of pathologic reports, fine needle aspiration, and ultrasonography or computed tomography were reviewed.

All 61 EFVPTC Hematoxylin and Eosin (H&E) slides of surgical specimens were blindly rereviewed according to the 2004 World Health Organization (WHO) classification of thyroid neoplasm by two pathologists (LHS and SEL). All 61 EFVPTC cases were further classified into two groups (NIFTP and I-EFVPTC) according to the consensus diagnostic criteria [6]. We categorized as I-EFVPTC, if cases with questionable/ incomplete capsular invasion. The pathologic T and N staging was determined according to the 8^th^ edition of the American Joint Committee on Cancer (AJCC) staging manual [23].

### Categorization of ultrasonography and fine-needle biopsy findings

The imaging findings of thyroid US were recorded according to K-TIRADS as; 1: no nodule; 2: benign: spongiform or partially cystic nodule with comet tail artifact or pure cyst; 3: low suspicion: partially cystic or iso-hyperechoic nodule without any of 3 suspicious US features (microcalcification, nonparallel orientation/ taller than wide, and speculated/ microlobulated margin); 4: intermediate suspicion; solid hypoechoic nodule without any of 3 suspicious US features or partially cystic or iso-hyperechoic nodule with any of 3 suspicious US features; and 5: high suspicion; solid hypoechoic nodule with any of 3 suspicious US features (21).

The FNA results were categorized into 6 groups according to the current TBSRTC as; Nondiagnostic or unsatisfactory; Benign; AUS/FLUS; FN/SFN; SM; and Malignant [12,13].

### Mutational analysis of the *BRAF* and *RAS* genes

Mutational analysis for *BRAF* V600E and K601E, *NRAS*, *HRAS*, and *KRAS* codons 12, 13 and 61, which are the most common sites of mutations, was performed. After slipping off the cover slips of FNA cytology slides, we dissect the target cells with a fine needle under the light microscope and use the in house ammonium sulfate DNA extraction method which was proven to be sensitive especially when handling specimens containing small number of cells atypical cells of interest were scraped and DNA was extracted [24]. Briefly, 20–50 μL of DNA extraction buffer solution (50 mM of Tris buffer, pH 8.3; 1 mM of EDTA, pH 8.0; 5% Tween 20; and 100 lg/mL of proteinase K) with 10% resin was added to the scraped cells and incubated at 56.8°C for a minimum of 1 h. After incubation, the tubes were heated to 100 ° C for 10 minutes, followed by centrifugation to pellet the debris, and 5 μL of the supernatant was used in the polymerase chain reaction (PCR). Purified PCR was done using a BioMix kit (Bioline, Taunton, MA). Extracted DNA was used as a template after quantification by NanoDrop (Thermo Scientific, Wilmington, DE). Primers were prepared to detect *BRAF* and *RA*S gene mutation sites (S1 Table). Each PCR mixture contained 0.4 pmol of forward and reverse primers, 0.2 mmoL of dNTP, 1.5 mmol/L of MgCl2, 1 · PCR buffer, 1.5 IU of Immolase DNA polymerase (Bioline, London, United Kingdom), and 5 μL of DNA in a total volume of 50 μL. Each PCR reaction contained 10 pmol/μL of primer set with one PCR buffer, 2.5 mM of MgCl_2_, and 1 μg of genomic DNA. The steps included initial denaturation (95°C) for 5 minutes, denaturation (94°C) for 30 seconds, annealing of the primers (52–57°C) to the DNA template for 30 seconds, and primer extension (72°C) for 30 seconds. All the above steps were repeated for 35 cycles followed by a final extension (72°C) for 5 minutes. After cooling to 4°C, final PCR products were confirmed by electrophoresis in a 2% agarose gel. To determine any genetic mutation, DNA sequencing was performed using the antisense primers. DNA sequences were then read using an analyzer (Bioedit version 7.2.0, Carlsbad, CA). Each sample was assayed twice in order to confirm the *BRAF* and *RAS* mutation status.

### Statistical analysis

We used the χ2 test and descriptive analysis to compare the rates of clinicopathological factors according to the presence or absence of capsular invasion. A *P*-value of less than 0.05 was considered to indicate a statistically significant difference. All analyses were carried out using SPSS version 22 (SPSS Inc, Chicago, IL).

## Results

### Patient demographics

A total of 61 patients with histologically confirmed EFVPTCs were enrolled in this study. The preoperative FNA results were available for all the patients. The clinicopathologic characteristics are presented in Table 1. The median age of patients was 45 years (range; 15–71 years). Thirty-two patients (53.5%) were <45 years of age and 29 patients (47.5%) were ≥45 years of age. The majority of patients received lobectomy (90.2%). Additional central lymph node dissection was performed for 32 (52.4%) patients. The median size of encapsulated FVPTC was 1.0 cm (range; 0.1–5 cm). Cervical lymph node metastases were identified in only one patient.

**Table 1:** Clinicopathological characteristics in NIFTP^a^ and I-EFVPTC^b^.

	Total (%) (n = 61)	NIFTP (%) (n = 20, 32.8%)	I-EFVPTC (%) (n = 41, 67.2%)	*P*-value
Sex				
Female	45	29 (70.7%)	16 (80.0%)	0.440
Male	16	12 (29.3%)	4 (20.0%)	
Age, years (range)	45 (15–71)	45 (17–74)	47 (15–71)	0.392
Tumor size, cm (range)	2.6 (0.7–8.7)	2.5 (0.5–6)	1.95 (0.7–8.7)	0.899
T_stage				
1	28 (45.9%)	16 (39.0%)	12 (60.0%)	0.083
2	21 (34.4%)	18 (43.9%)	3 (15.0%)	
3	12 (19.7%)	7 (17.1%)	5 (25.0%)	
N_stage				0.767
0	31 (50.8%)	10 (95.5%)	21 (51.2%)	
1	1 (1.6%)	0	1 (2.4%)	
NA^c^	29 (47.5%)	10 (50.0%)	19 (46.3%)	
Operation				0.559
Lobectomy	55 (90.2%)	19 (95.0%)	36 (87.8%)	
Subtotal thyroidectomy	2 (3.3%)	0	2 (4.9%)	
Total thyroidectomy	4 (6.6%)	1 (5.0%)	3 (7.3%)	

Abbreviations:

^a^NIFTP, non-invasive follicular thyroid neoplasm with papillary-like nuclear features;

^b^I-EFVPTC, invasive encapsulated follicular variant of papillary thyroid carcinoma;

^c^NA, not applicable

**Fig 1:**
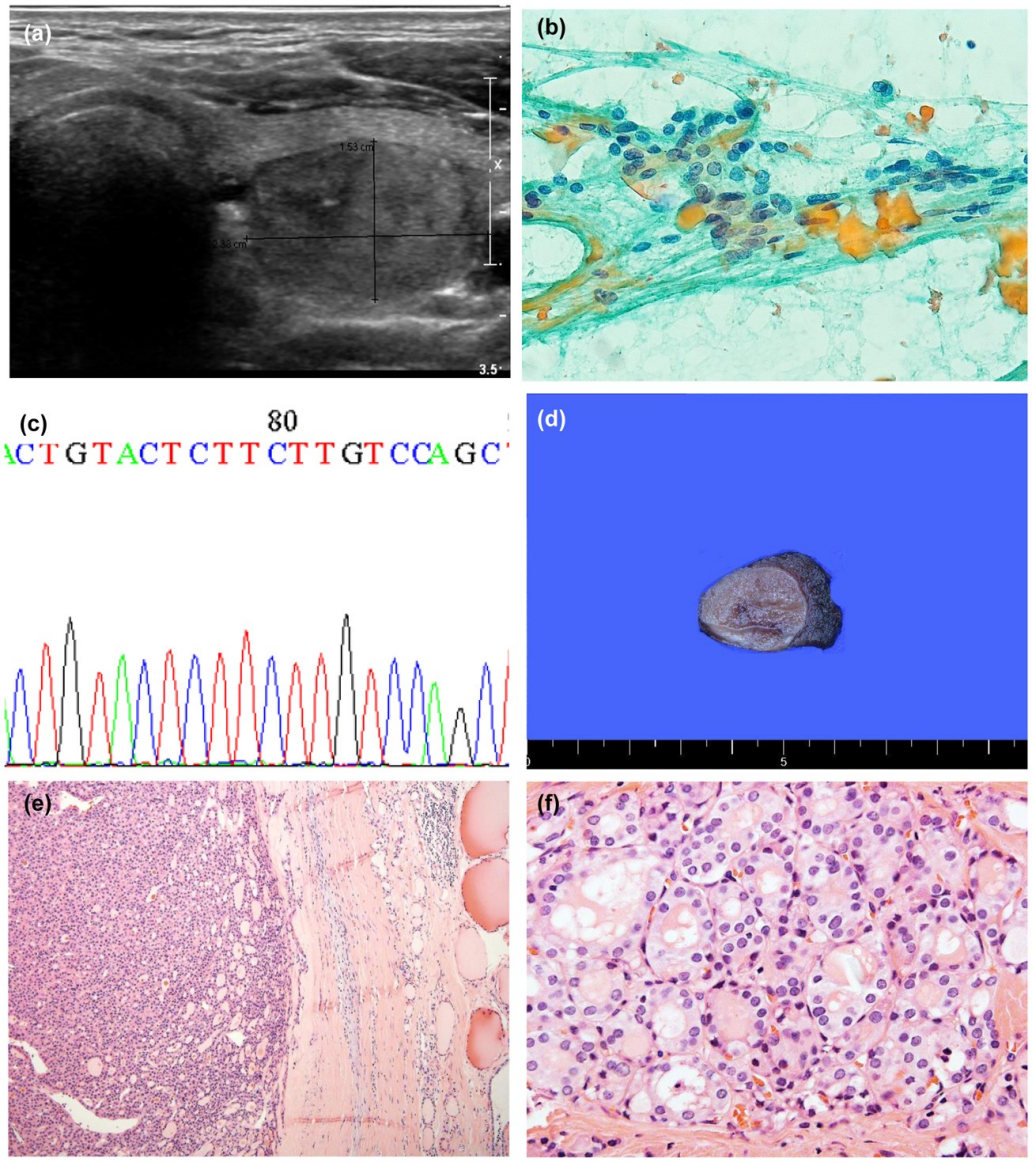
Representative case of non-invasive follicular thyroid neoplasm with papillary-like nuclear features (NIFTP). (a) Ultrasonographic image shows a 2.4 cm sized intermediate suspicious lesion (K-TIRADS 4). (b) Fine needle aspiration cytology shows follicular cells with mild nuclear atypia, including nuclear enlargement, and pale chromatin. This lesion was diagnosed as AUS/FLUS. (c) Gross examination shows an encapsulated gelatinous mass. (d) No *NRAS* mutation was identified in this lesion. (e) At low magnification, microscopic examination reveals a well-capsulated follicular tumor without capsular invasion. (f) Tumor cells show mild nuclear atypia, including nuclear enlargement, nuclear membrane irregularity, pale chromatin, and nuclear grooves. This was diagnosed as NIFTP.

**Fig 2:**
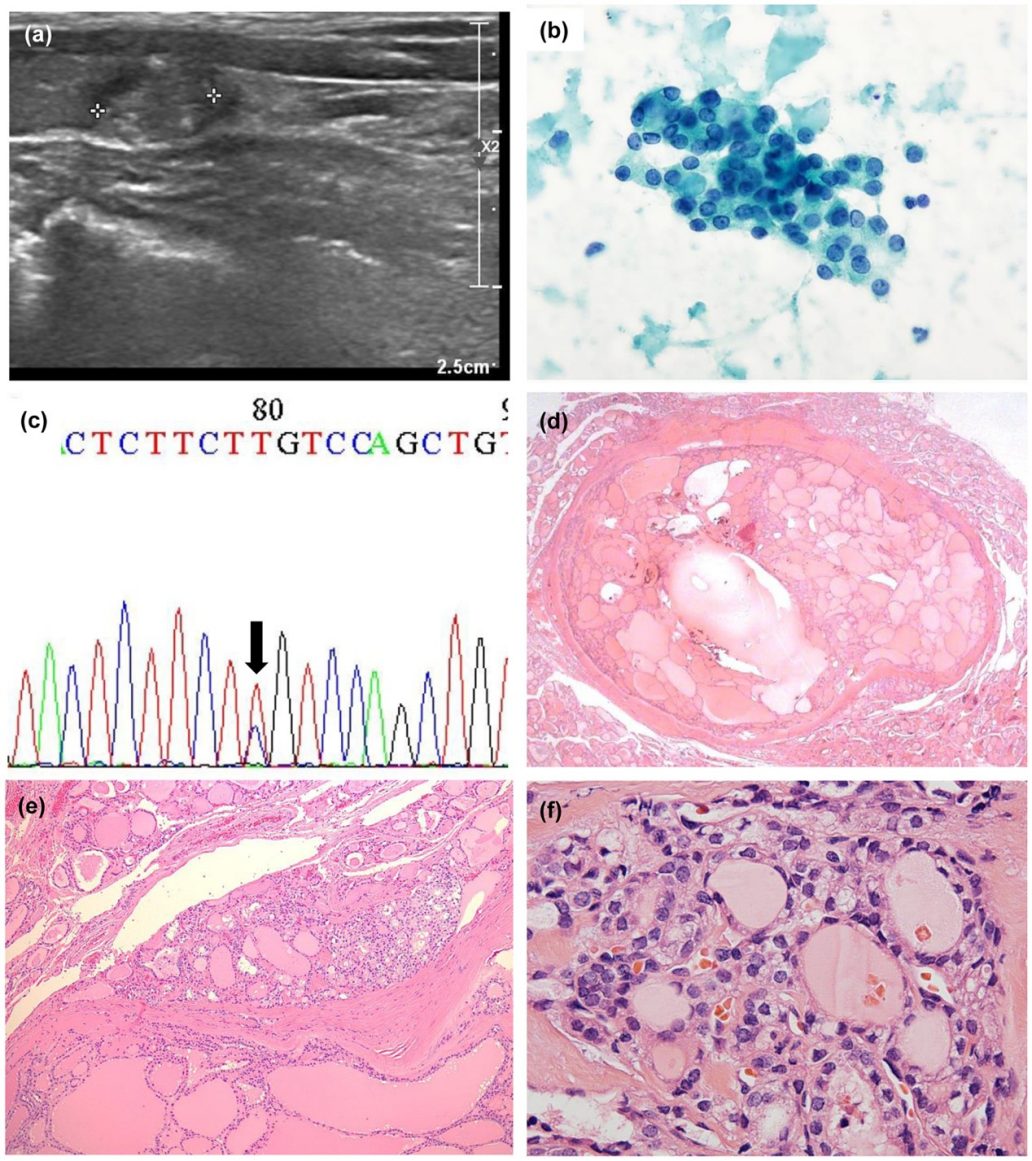
Representative case of an invasive encapsulated follicular variant of papillary thyroid carcinoma (I-EFVPTC). (a) Ultrasonographic image shows a 0.8 cm sized nodule with rim and internal calcification (K-TIRADS 5). (b) Fine needle aspiration cytology shows some follicular cells with mild to moderate nuclear atypia, including nuclear enlargement, nuclear membrane thickening, and pale chromatin. This lesion was diagnosed as TBRSTC V (suspicious for malignancy). (c) *NRAS* Q61R mutation was identified in this lesion. (d-e) At low magnification, microscopic examination reveals a well-capsulated follicular tumor with capsular invasion. (f) Tumor cells show nuclear atypia, including nuclear enlargement, nuclear membrane irregularity, pale chromatin, and nuclear grooves. This was diagnosed as I-EFVPTC.

EFVPTC was subdivided into two groups according to the consensus diagnostic criteria [6] as NIFTP and I-EFVPTC, representative clinicopathologic and molecular features of each two group are described in Figs 1 and 2. Twenty patients (32.8%) and 41 patients (67.2%) were diagnosed with NIFTP and I-EFVPTC, respectively. One but all patients in NIFTP subgroup underwent lobectomy. Only one patient with I-EFVPTC had cervical LN metastasis. There were no significant differences in age, sex, and median tumor size between NIFTP and I-EFVPTC subgroups. Also, there was no recurrence in both groups during a median of 12 months (1–41 months) of follow-up.

### Image analysis

Based on the detailed image analyses (Table 2), the predominant US features of NIFTP were identified as parallel orientation (100%), smooth margin (100%), hyper-/isoechogenicity (60%), solid or almost completely solid (90%), and no calcification (70%). The most common findings of I-EFVPTC on the US were a parallel orientation (100%), smooth margin (90.2%), hyper-/isoechogenicity (51.2%), solid or almost completely solid (78%), and no calcification (51.2%). The US features between the two groups were not significantly different.

**Table 2:** Ultrasonographic findings of patients with NIFTP^a^ and I-EFVPTC^b^.

Characteristics	Total (%) (n = 61)	NIFTP (%) (n = 20, 32.8%)	I-EFVPTC (%) (n = 41, 67.2%)	*P*-value
**Orientation**				NA^c^
Taller-than-wide/ Non-parallel	0 (0)	0 (0)	0 (0)	
Wider-than-tall/Parallel	61 (100)	20 (100)	41 (100)	
**Margin**				0.148
Smooth / Ill-defined	57 (93.4)	20 (100.0)	37 (90.2)	
Lobulated or irregular	4 (6.6)	0 (0)	4 (9.8)	
**Echogenicity**				0.801
Anechoic	0 (0)	0 (0)	0 (0)	
Hyper-/ isoechoic	33 (54.1)	12 (60.0)	21 (51.2)	
Hypoechoic	24 (39.3)	7 (35.0)	17 (41.5)	
Very hypoechoic	4 (6.6)	1 (5.0)	3 (7.3)	
**Composition**				0.543
Cystic or almost completely cystic/ Spongiform	1 (1.6)	0 (0)	1 (2.4)	
Mixed cystic and solid	10 (16.4)	2 (10.0)	8 (19.5)	
Solid or almost completely solid	50 (82.0)	18 (90.0)	33 (78.0)	
**Calcification**				0.295
No calcification	35 (57.4)	14 (70.0)	21 (51.2)	
Macrocalcifications	11 (18.0)	2 (10.0)	9 (22.0)	
Peripheral (rim) calcifications	7 (11.5)	3 (15.0)	4 (9.8)	
Punctate echogenic foci	8 (13.1)	1 (5.0)	7 (17.1)	

Abbreviations:

^a^NIFTP, non-invasive follicular thyroid neoplasm with papillary-like nuclear features;

^b^I-EFVPTC, invasive encapsulated follicular variant of papillary thyroid carcinoma;

^c^NA: not available

Preoperative neck US results were categorized into four category (benign, low suspicion, intermediated suspicion, and high suspicion) according to the K-TIRADS. By applying K-TIRADS, K-TIRADS 3 was identified as the most common preoperative sonographic diagnosis (29 patients, 47.5%). In NIFTP subgroup, K-TIRADS 3 was observed in 11 patients (55.0%) and K-TIRADS 4 in 9 patients (45.0%). There was no K-TIRADS 2 and K-TIRADS 5 in NIFTP subgroup. In I-EFVPTC subgroup, K-TIRADS 3 was observed in 18 patients (43.9%), K-TIRADS 4 in 16 patients (39.0%), K-TIRADS 5 in 6 patients (14.6%), and K-TIRADS 2 in one patient (2.4%). There was no significant difference in the distribution of category between the NIFTP and I-EFVPTC subgroups (*P* = 0.25) (Table 3).

**Table 3:** K-TIRADS and TBSRTC category in NIFTP^a^ and I-EFVPTC^b^.

Classification systems	Category	Total (%) (n = 61)	NIFTP (%) (n = 20, 32.8%)	I-EFVPTC (%) (n = 41, 7.2%)	*P*-value
K-TIRADS^c^	1: No nodule	0 (0)	0 (0)	0 (0)	0.25
	2: Benign	1 (1.6)	0 (0)	1 (2.4)	
	3: Low suspicion	29 (47.5)	11 (55.0)	18 (43.9)	
	4: Intermediate suspicion	25 (41.0)	9 (45.0)	16 (39.0)	
	5: High suspicion	6 (9.8)	0 (0)	6 (14.6)	
TBSRTC^d^	I: Nondiagnostic	2 (3.3)	1 (5.0)	1 (2.4)	0.36
	II: Benign	4 (6.6)	1 (5.0)	3 (7.3)	
	III: AUS/FLUS^e^	21 (34.4)	4 (20.0)	17 (41.5)	
	IV: FN/SFN^f^	16 (26.2)	8 (40.0)	8 (19.5)	
	V: SM^g^	18 (29.5)	6 (30.0)	12 (29.3)	
	VI: Malignant	0 (0)	0 (0)	0 (0)	

Abbreviations:

^a^NIFTP, non-invasive follicular thyroid neoplasm with papillary-like nuclear features;

^b^I-EFVPTC, invasive encapsulated follicular variant of papillary thyroid carcinoma;

^c^K-TIRADS, Korean Thyroid Imaging Reporting and Data System;

^d^TBSRTC, The current Bethesda System for Reporting Thyroid Cytopathology;

^e^AUS/FLUS, Atypia of undetermined significance or follicular lesion of undetermined significance;

^f^FN/SFN, Follicular neoplasm or suspicious for a follicular neoplasm;

^g^SM, Suspicious for Malignancy.

### Cytopathology analysis

The cytologic analysis was performed according to the Bethesda System for Reporting Thyroid Cytopathology (TBSRTC) [20]. The majority of the EFVPTC were classified as indeterminate TBSRTC category (55 patients, 90.2%). Of them, the AUS/FLUS category was identified as the most common preoperative cytopathological diagnosis (21 patients, 34.4%). All patients with AUS/FLUS category showed cytologic atypia, 12 showed architectural atypia when AUS/FLUS category was divided into those with either cytologic or architectural atypia according to the definition of TBRSTC. Eighteen patients (29.5%) were categorized as SM and 16 patients (26.2%) as FN/SFN. Four patients (6.6%) were categorized as benign. In NIFTP subgroup, 60.0% and 30.0% of patients were preoperatively diagnosed as AUS/FLUS or FN/SFN and SM category. In I-EFVPTC subgroup, 61.0% and 29.3% of patients were preoperatively diagnosed as AUS/FLUS or FN/SFN and SM category. There was no statistical significance in both of cytological and architectural atypia in AUS/FLUS category between NIFTP and I-EFVPTC subgroups. Notably, there was no malignant category in both NIFTP and I-EFVPTC subgroups. TBSRTC category were indistinguishable between NIFTP and I-EFVPTC subgroups (*P* = 0.36) (Table 3).

### Molecular analysis

Genetic alterations were observed in 30 (49.1%) of 61 EFVPTCs. *BRAF* and *RAS* mutations were detected in 6.6% and 42.6% of 61 EFVPTCs, respectively. Thirty-one cases (50.8%) were wild-type *BRAF* and *RAS*. Table 4 shows the molecular genotyping of the NIFTP and I-EFVPTC. *BRAF* mutations were found in 0% and 9.8% of NIFTP and I-EFVPTC, respectively. In I-EFVPTC, there were three *BRAF* V600E mutations and one *BRAF* K601E mutation. There was no significant difference between the two groups in the frequency of *BRAF* mutations (*P* = 0.293). *RAS* mutations were found in 5 (25.0%) and 21 (51.2%) cases of NIFTP and I-EFVPTC, respectively. *NRAS* mutation in codon 61 was the most common mutation in NIFTP (25%) and I-EFVPTC (36.6%). In NIFTP, only *NRAS* mutations were present in codon 61. The most common genotyping of *RAS* mutation in NIFTP was *NRAS* Q61R (n = 3, 15.0%), followed by *NRAS* Q61K (n = 1, 5.0%), and *NRAS* Q61L (n = 1, 5.0%). The most common genotyping of *RAS* mutation in I-EFVPTC was *NRAS* Q61R (n = 12, 29.3%), followed by *HRAS* Q61K (n = 3, 7.3%), and *NRAS* Q61K (n = 2, 4.9%). *NRAS* G12V, *HRAS* G13R, and *KRAS* Q61R mutations were detected in 1 (2.4%), 1 (2.4%), and 1 (2.4%) I-EFVPTC, respectively. There were no *HRAS* Q61R and *KRAS* codon 12/13 mutations in the two groups. The frequency of *RAS* mutation differed between the two groups, although there was a marginally significant difference (*P* = 0.052). The frequency of *RAS* mutation in I-EFVPTC was twice as high as that of NIFTP. Interestingly, wild-type *BRAF* and *RAS* were significantly more commonly observed in NIFTP than I-EFVPTC subgroup (75.0% vs. 39.0%, *P* = 0.008).

**Table 4:** Molecular analysis of *BRAF* and *RAS* in NIFTP^a^ and I-EFVPTC^b^.

	Total (n = 61)	NIFTP (n = 20, 32.8%)	I-EFVPTC (n = 41, 67.2%)	*p-value*
***BRAF***				0.293
Wild	57 (93.4%)	20 (100%)	37 (90.2%)	
Mutant	4 (6.6%)	0	4 (9.8%)	
V600E	3 (4.9%)	0	3 (7.3%)	
K601E	1 (1.6%)	0	1 (2.4%)	
***RAS***				0.052
Wild	35 (57.4%)	15 (75.0%)	20 (48.8%)	
Mutant	26 (42.6%)	5 (25.0%)	21 (51.2%)	
*NRAS* Q61R	15 (24.6%)	3 (15.0%)	12 (29.3%)	
*NRAS* Q61K	3 (4.9%)	1 (5.0%)	2 (4.9%)	
*NRAS* Q61L	2 (3.3%)	1 (5.0%)	1 (2.4%)	
*NRAS* G12V	1 (1.6%)	0	1 (2.4%)	
*HRAS* Q61K	3 (4.9%)	0	3 (7.3%)	
*HRAS* Q61R	0	0	0	
*HRAS* G13R	1 (1.6%)	0	1 (2.4%)	
*KRAS* Q61R	1 (1.6%)	0	1 (2.4%)	
*KRAS* codon 12/13	0	0	0	
***BRAF/RAS***				0.008*
Wild	31 (50.8%)	15 (75.0%)	16 (39.0%)	
Mutant	30 (49.2%)	5 (25.0%)	25 (61.0%)	

Abbreviations:

^a^NIFTP, non-invasive follicular thyroid neoplasm with papillary-like nuclear features;

^b^I-EFVPTC, invasive encapsulated follicular variant of papillary thyroid carcinoma

*Statistically significant with P-value <0.05

## Discussion

Recently, an international and multidisciplinary study suggested a reclassification of the noninvasive EFVPTC, as NIFTP due to the indolent biologic behavior for long term follow-up to reduce overtreatment of this group [6]. The current paradigm in the treatment of patients with NIFTP is limited surgery such as lobectomy rather than subtotal/complete thyroidectomy or postoperative radioiodine treatment. Therefore, comprehensive evaluation for the diagnosis of preoperative NIFTP is important for clinicians to achieve better decision to manage the patients.

EFVPTC and IFVPTC are biologically and molecularly distinct subgroups [25]. IFVPTC is rather close to classic PTC in terms of clinical and biologic characteristics [4,26]. IFVPTC showed a significantly higher rate of K-TIRADS 5 category than other FVPTCs on the US [11,20]. IFVPTC was mainly diagnosed as SM or malignant category based on cytological analysis [27,28]. Furthermore, IFVPTCs have a significantly higher *BRAF* V600E mutation and lower *RAS* mutation rate compared with EFVPTC. Therefore, it is easier to differentiate preoperative IFVPTC from FVPTCs than distinguishing between preoperative NIFTP and I-EFVPTC. In this study, IFVPTC has been excluded from histologic subtypes of FVPTCs for the purpose of analysis. Therefore, we aimed to distinguish the histologically confirmed NIFTP from I-EFVPTC based on preoperative radiologic, cytological, and molecular features.

Ultrasonography (US) features of FVPTCs include relatively benign appearance compared to the typical US features seen in classic PTCs [29,30]. The common US features of FVPTC are a solid composition, hypoechogenicity, microlobulated margins, absence of calcification, and a parallel orientation [29,30]. However, differentiation of the characteristics of NIFTP from I-EFVPTC based on preoperative US features remains uncertain. In our study, K-TIRADS 3 was the most common preoperative sonographic diagnosis in both NIFTP and I-EFVPTC groups. Although there was no significant difference between the two groups, K-TIRADS 5 classification in NIFTP, unlike I-EFVPTC group, was not identified. Hahn et al. [20] recently reported that non-NIFTP lesions were diagnosed as a significantly higher rate of K-TIRADS 5 on the US compared with NIFTP lesions. Furthermore, a recent study from Korea showed that US findings of the FVPTCs differed significantly according to tumor invasiveness [11]. Furthermore, tumor invasiveness showed a significant positive correlation with K-TIRADS. Most of the common subtypes were K-TIRADS 3 nodules in NIFTP, K-TIRADS 4 nodules in I-EFVPTC, and K-TIRADS 5 nodules in I-FVPTC [11].

Since the introduction of TBSRTC in 2007, it has been widely used as the diagnostic classification for the FNA as a uniform reporting system which facilitated effective communication among variable groups and cytologic-histologic correlation for thyroid diseases [13]. In 2017, TBSRTC was revised for reporting the thyroid cytology guidelines for the management of patients with thyroid nodules [12]. The important change incorporated about NIFTP in the 2017 update is the definition and diagnostic criteria for FN/SFN (category IV) in light of the new entity of NIFTP [12]. Follicular-patterned cases with mild nuclear changes (enlargement of size, membrane irregularity, and/or pale chromatin) can be classified as FN/SFN so long as definitive features of classic PTC such as true papillae and intranuclear pseudoinclusions are absent. This change of definition may include NIFTP or I-FVPTC in FN/SFN category [12]. In our study, 90% of NIFTP cases and 89.8% of I-EFVPTC cases were classified as indeterminate category of TBSRTC similar to previous results. Especially, AUS/FLUS were the most common preoperative cytopathological diagnosis in both NIFTP and I-EFVPTC groups. It is significant worth mentioning that none of the cases were classified as a malignant category in both NIFTP and I-EFVPTC groups. In previous studies, a variable percentage of NIFTP cases (0–18%) have been classified as a malignant category [16,17,31]. Unfortunately, we did not identify the difference in distribution in TBSRTC category between NIFTP and I-EFVPTC subgroups. These findings are in agreement with a majority of the published data [5,16,32]. Meanwhile, the differences in the detailed cytomorphologic findings between the two groups were reported [33]. In the case of comparison between NIFTP and I-EFVPTC subgroups, a predominant microfollicular pattern and nuclear groove were more likely to be associated with NIFTP [33]. However, the difference in distribution in TBSRTC category between NIFTP and I-EFVPTC could not be identified in the reported study. As a result, cytology alone could not be considered reliable for the differentiation between NIFTP and I-EFVPTC.

It was reported that the absence of *BRAF* V600E mutation in NIFTP is a characteristic feature [25,26], whereas the rate of *BRAF* V600E mutation was variously reported in I-EFVPTC (0~30%) [25,34,35]. In the present study, *BRAF* mutation was not found in NIFTP, but present in 9.8% of cases in I-EFVPTC as expected. Meanwhile, *RAS* mutations were detected in 42.6% of 61 EFVPTCs. The proportion of *RAS* mutations differed according to the invasiveness even though marginal significance was noted (*P* = 0.052). The frequency of *RAS* mutation in I-EFVPTCs (51.2%) was twice as high as that of NIFTP (25%). Therefore, wild-type *BRAF* and *RAS* were significantly more commonly observed in NIFTP subgroup than I-EFVPTC subgroup. The obtained results are rather inconsistent with that of recent studies [22,34,36,37], which did not distinguish between NIFTP and I-EFVPTC based on *RAS* mutation. Kim et al. [37] reported that the frequency of *RAS* mutation in I-EFVPTCs (66.7%) was higher than NIFTP (48.6%), but there was no significant difference. Furthermore, it was reported that wild-type *BRAF*, *RAS*, and *RET* in NIFTP were significantly higher than in I-EFVPTC subgroup. It has been reported that the proportion of *RAS* mutation in NIFTP was rather higher than in I-EFVPTCs [22,34,36]. Song et al. [36] recently reported that molecular profiles including transcriptomic profiles were comparable in NIFTP, I-EFVPTC, and follicular adenoma/minimally invasive follicular carcinoma, thus the profiles of NIFTP and I-EFVPTC were not different from each other. This discrepancy might be attributed to different diagnostic criteria used to classify EFVPTC. Intra- and inter-observer variation to the presence of capsular or vascular invasion has been reported even among experienced pathologists [34,38,39]. We applied the rigid standard of capsular or vascular invasion and excluded samples with questionable or incomplete capsular invasion from NIFTPs. Furthermore, a recent study demonstrated that the arbitrary cutoff of 1% papillae has resulted in the misclassification of classic PTCs with predominant follicular architecture as NIFTPs [34]. Therefore, more strict criteria were used for the cutoff for 0% true papillae in our study. As a consequence, relatively low incidence (32.8%) of NIFTP was observed in this study compared with other studies (40–67.9%) [22,34,36,37]. Therefore, the difference in frequency of *RAS* mutation in the two groups could be rather controversial. Further studies are needed to determine whether the frequency of *RAS* mutation could be helpful in discriminating between NIFTP and I-EFVPTC.

The limitation of this study was its retrospective study design regarding the diagnosis of NIFTP. Although the majority of the tumor capsule and tumor parenchyma were examined, the problem is the possibility of misdiagnosed cases due to lack of ideal condition to inspect of the whole tumor capsule in retrospective study.

We tried to find out possible diagnostic clues to differentiate between NIFTP and I-EFVPTC using preoperative radiologic, cytopathologic, and molecular findings. None of the cases were classified as K-TIRADS 5 (high suspicion) in NIFTP contrary to I-EFVPTC based on the US. In the molecular analysis, the frequency of *RAS* mutation in I-EFVPTCs was as high as that of NIFTP and wild-type *BRAF* and *RAS* was significantly high in NIFTP. Unfortunately, there exist overlapping features between the two groups in clinical practice, which precludes conclusive distinction between the radiology, cytology, and molecular analysis.

In conclusion, the diagnosis of NIFTP based on comprehensive analysis including preoperative radiologic, cytologic, and molecular analysis was not confirmable but could perceive or at least favor the diagnosis of NIFTP.

## Supporting information

S1 Table Nucleotide sequences of primers used for direct sequencing. (DOCX)
