# Supplementary material for: Comprehensive analysis for diagnosis of preoperative non-invasive follicular thyroid neoplasm with papillary-like nuclear features
Source: PLoS One. 2019 Jul 5;14(7):e0218046. doi: 10.1371/journal.pone.0218046 (PMC6611592; doi:10.1371/journal.pone.0218046)
Supplement: S1 Table — (DOCX) [file pone.0218046.s001.docx]

**S1 Table. Nucleotide sequences of primers used for direct sequencing.**

|  | Codon | Primers | Length (mer) | Amplicon (bp) | Nucleotide sequence | Temperature (Celsius) |
| --- | --- | --- | --- | --- | --- | --- |
| *BRAF* | 600/601 | UP |  | 180 | AACTCTTCATAATGCTTGCTCTGA | 56 |
|  |  | LP |  |  | CAGACAACTGTTCAAACTGATGGGACC |  |
| *NRAS* | 61 | Forward | 20 | 149 | GTGAAACCTGTTTGTTGGAC | 56 |
|  |  | Reverse | 20 |  | CCTGTAGAGGTTAATATCCG |  |
|  | 12/13 | Forward | 21 | 176 | CTTGCTGGTGTGAAATGACTG | 53-57 |
|  |  | Reverse | 20 |  | TCCGACAAGTGAGAGACAGG |  |
| *HRAS* | 61 | Forward | 19 | 154 | GGAGACGTGCCTGTTGGAC | 56 |
|  |  | Reverse | 20 |  | GGTTCACCTGTACTGGTGGA |  |
|  | 12/13 | Forward | 19 | 129 | CTGAGGAGCGATGACGGAA | 55-58 |
|  |  | Reverse | 20 |  | AGGCTCACCTCTATAGTGGG |  |
| *KRAS* | 61 | Forward | 21 | 135 | GTTTCTCCCTTCTCAGGATTC | 56 |
|  |  | Reverse | 18 |  | CCCTCCCCAGTCCTCATG |  |
|  | 12/13 | Forward | 21 | 165 | AAGGCCTGCTGAAAATGACTG | 60-65 |
|  |  | Reverse | 22 |  | GGTCCTGCACCAGTAATATGCA |  |
